# Development of a Compound Speckle Interferometer for Precision Three-Degree-of-Freedom Displacement Measurement

**DOI:** 10.3390/s21051828

**Published:** 2021-03-05

**Authors:** Hung-Lin Hsieh, Bo-Yen Sun

**Affiliations:** Department of Mechanical Engineering, National Taiwan University of Science and Technology, No. 43, Keelung Road, Section 4, Da’an District, Taipei 106335, Taiwan; tempest051@gmail.com

**Keywords:** speckle interferometer, displacement, heterodyne interferometry

## Abstract

In this study, a compound speckle interferometer for measuring three-degree-of-freedom (3-DOF) displacement is proposed. The system, which combines heterodyne interferometry, speckle interferometry and beam splitting techniques, can perform precision 3-DOF displacement measurements, while still having the advantages of high resolution and a relatively simple configuration. The incorporation of speckle interferometry allows for non-contact displacement measurements by detecting the phase of the speckle interference pattern formed from the convergence of laser beams on the measured rough surface. Experiments were conducted to verify the measurement capabilities of the system, and the results show that the proposed system has excellent measurement capabilities suitable for future real-world applications.

## 1. Introduction

In recent years, the rapid development of micro-manufacturing and related industries has led to an increase in demand for precision measurement technologies with long range, high precision and good versatility. For example, precise, long range positioning ensures uniform and stable processing during the photolithographic procedure, which greatly improves the production quality and yield [1]. Another important quality for precision measurement technologies is non-contact measurement capability, that is the ability to measure displacement or movement without requiring physical contact with the sample, so as not to risk altering or damaging it [2].

A number of non-contact measurement technologies have been developed, including capacitive displacement sensors, linear encoders, fiber optic sensors, and interferometers, etc., each with their own advantages and limitations. For example, although capacitance displacement sensors typically have a nanometer-level measurement resolution, their measurement range can only reach the micron level, restricting them to small displacement measurement applications [3]. Meanwhile, linear encoders also have a high measurement resolution, but also have a long measurement range. The problem is that most linear encoders are only capable of measuring a single degree-of-freedom (1-DOF), thus limiting their application potential [4]. In contrast, laser interferometers are already widely used in precision metrology applications because of their high resolution, high sensitivity, fast response, and long measurement range [5]. Although laser interferometers generally perform well for a wide range of applications, most can only perform 1-DOF measurement without changing the structure of the configuration of the optical path. If multi-DOF measurement is required, it can only be achieved by altering the existing configuration or by linking multiple sets of interferometer systems [6]. This not only greatly complicates the system configuration, but the build-up of misalignments and positioning inaccuracies tends to have a negative effect on measurement results. Therefore, the development of a long-range laser interferometer system with non-contact measurement capability, which also possesses good versatility for simultaneous multi-DOF displacement measurement is of great importance.

In response to this need, this study presents a compound speckle interferometer developed by incorporating heterodyne interferometry, speckle interferometry and beam-splitting techniques, allowing for high accuracy, high resolution, non-contact measurement. The system includes of a heterodyne laser light source, two different types of speckle-based optical configurations and a phase demodulation system. The heterodyne light source is generated by a He-Ne laser and an electro-optical modulator (EOM), which can filter out low-frequency disturbances, improving measurement stability. The light then passes into a compound speckle optical configuration comprising three different speckle optical configurations, one symmetrical and two asymmetric. Each optical configuration consists of two beams directed onto a single point on the measured surface and scattered. In accordance with the speckle effect, the overlapping of the scattered light from the two beams forms a speckle pattern, the signal intensity of which is acquired by a photodetector. When displacement occurs on the measured surface, there is a corresponding phase shift introduced into the signal. The displacement information can be derived from the detected phase shift by analyzing the data using our self-developed phase demodulation program. The symmetricity of the beams relative to the axis perpendicular to the measured surface dictates the measurement capability of the speckle measurement system, with a symmetrical configuration allowing for the measurement of in-plane (IP) displacement while an asymmetrical design is able to measure both IP and out-of-plane (OP) motion, although not simultaneously. The combination of a symmetrical and asymmetrical speckle optical configuration means that the system is able to measure both IP and OP displacement simultaneously. The proposed system is also non-contact by nature, thus effectively overcoming the limitations to measurement range imposed on traditional interferometers by the size of their component mirrors or gratings, and giving the system a long measurement range for all displacements.

In order to verify the measurement capability of the proposed compound speckle interferometer, several experiments were conducted using commercially available precision displacement stages. The measurement results were compared with data obtained from the built-in capacitance sensor on the stage, verifying the high accuracy, high resolution displacement measurement on sample surfaces of this measurement system. The system design enables long range measurement for a minimum of 3-DOF, while having a relatively simple configuration that can be easily assembled, calibrated and adjusted, allowing for great utility and versatility.

## 2. Measurement Principles

In this section, the relationship between the phase variation of the speckle interference signal resulting from the in-plane (IP) and out-of-plane (OP) displacement of a moving non-mirror surface is explained. Specifically, the basic relation between the phase variation and displacement is established, and the principles for measurement using laser speckle interferometry of the two types of variation based on two special cases are explained in detail. Finally, the design concepts used to construct the proposed interferometer and the utilization of the measurement principles are described.

### 2.1. Relation between Phase Variation and Displacement in Speckle Interferometry

According to the theory behind the phenomenon of speckle intensity patterns, when a laser beam is projected onto a non-mirror surface, it is scattered in all directions, forming a hemisphere of subjective speckles. The proposed system directly captures a small portion of the overlapping speckle patterns reflected off a rough surface, which are subjective speckles by definition. When two such hemispheres overlap, a speckle intensity pattern will form. Observation and analysis of changes in the speckle pattern allow measurement of the surface displacement and profile.

**Figure 1:**
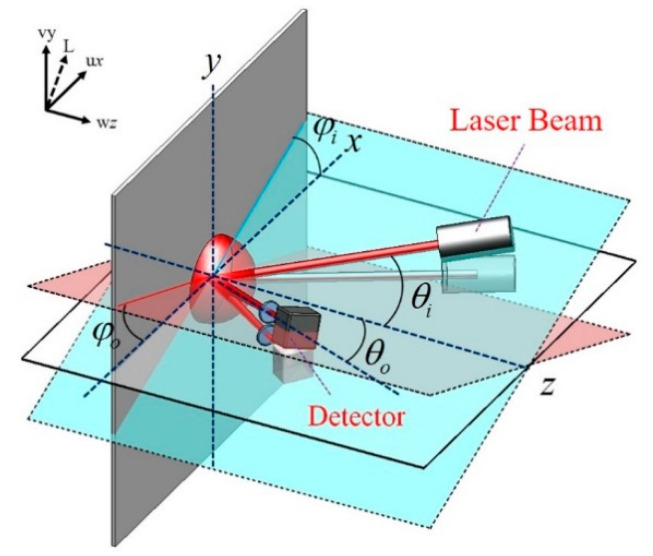
Basic configuration of laser speckle interferometer for measuring surface displacement.

As shown in Figure 1, the combined IP and OP displacement *L* of a surface can be represented in vector form as follows:

(1) L=ux+vy+wz,

where **u** and **v** represent the IP displacement on the *x*- and *y*-axis, respectively, and **w** the OP displacement on the *z*-axis, while *x*, *y* and *z* represent the respective unit vectors. For a single laser beam, the relationship between the optical phase variation (δ) of the scattered light and displacement can be written as follows [7]:

(2) $$\;\delta =\frac{2\pi }{\lambda }\left[u\left(\sin\theta_{i}\cos\varphi_{i}-\sin\theta_{o}\cos\varphi_{o}\right)+v\left(\sin\theta_{i}\sin\varphi_{i}-\sin\theta_{o}\sin\varphi_{o}\right)+w\left(\cos\theta_{i}+\cos\theta_{o}\right)\right],$$

where *λ* is the wavelength of the laser source, *θ_i_* is the incidence angle and *θ_o_* is the observation angle relative to the normal axis *z*, while *φ_i_* and *φ_o_* are the angles of the planes of incidence and observation relative to the horizontal plane *xz*, respectively. The equation clearly shows that the optical phase variation of a speckle beam is influenced by the angles of incidence and observation.

If two or more laser beams of the same wavelength *λ* converge on a point on a surface, a speckle interference pattern will be formed [8]. The phase variation difference Φ of the combined heterodyne interference pattern formed by two beams can be written as follows:

(3) $$\begin{array}{l} \Phi =\delta_{1}-\delta_{2}=\frac{2\pi }{\lambda }[u\left(\sin\theta_{i1}\cos\varphi_{i1}-\sin\theta_{i2}\cos\varphi_{i2}-\sin\theta_{o1}\cos\varphi_{o1}+\sin\theta_{o2}\cos\varphi_{o2}\right)\\ \;+v\left(\sin\theta_{i1}\sin\varphi_{i1}-\sin\theta_{i2}\sin\varphi_{i2}-\sin\theta_{o1}\sin\varphi_{o1}+\sin\theta_{o2}\sin\varphi_{o2}\right)\\ \;+w\left(\cos\theta_{i1}-\cos\theta_{i2}+\cos\theta_{o1}-\cos\theta_{o2}\right)], \end{array}$$

where *δ*_1_, *δ*_2_ are the phase variations of the two beams, *θ_i_*_1_, *θ_i_*_2_, *θ_o_*_1_, and *θ_o_*_2_ are their incident and observation angles, and *φ_i_*_1_, *φ_i_*_2_, *φ_o_*_1_, and *φ_o_*_2_ are the angles of the planes of incidence and observation relative to plane *xz*. Now, if the two beams are incident on a single point, and a single sensor is used to receive the signals from both beams, both observation angle values will be the same, i.e., *θ_o_*_1_ = *θ_o_*_2_ and *φ_o_*_1_ = *φ_o_*_2_, reducing Equation (3) to:

(4) $$\Phi =\frac{2\pi }{\lambda }[u\left(\sin\theta_{i1}\cos\varphi_{i1}-\sin\theta_{i2}\cos\varphi_{i2}\right)+v\left(\sin\theta_{i1}\sin\varphi_{i1}-\sin\theta_{i2}\sin\varphi_{i2}\right)+w\left(\cos\theta_{i1}-\cos\theta_{i2}\right)].$$

Thus, removing the influence of the observation angle. The above equation will be used as the general case formula of speckle phase variation for further calculations in this paper.

**Figure 2:**
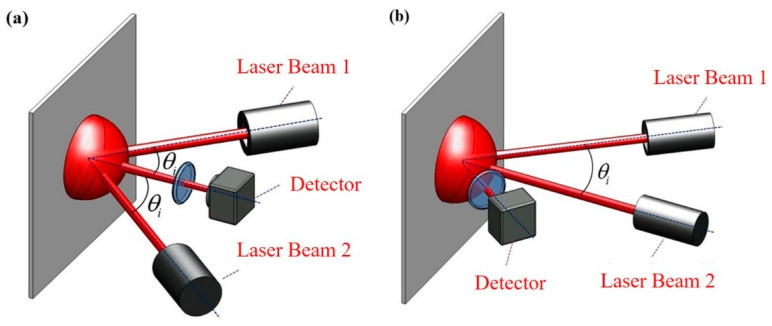
(**a**) Symmetrical speckle interferometry; (**b**) Asymmetric speckle interferometry.

As shown in Figure 2a, for a symmetrical speckle interferometer, assuming that the incident beams are symmetrically angled, the incident angles of the beams will have the same value but the opposite sign, that is *θ_i_*_1_ = −*θ_i_*_2_ = *θ_i_*. Assuming that both beams lie on the *xz*-plane (*φ_i_*_1_ = *φ_i_*_2_ = 0), then Equation (4) becomes:

(5) $$\Phi =u\frac{4\pi }{\lambda }\sin\theta_{i}.$$

This relation can then be used to calculate the IP displacement **u** from the measured phase variation difference, while the IP displacement on the *y*-axis **v** can be obtained in a similar manner. Note that the OP displacement **w** has no effect on the phase variation difference for this type of speckle interferometer.

For an asymmetric speckle interferometer, assume that one of the incident beams is normal to the measured surface (*θ_i_*_1_ = 0), as shown in Figure 2b, while the other beam is at an arbitrary angle (*θ_i_*_2_ = *θ_i_*), once again assuming both beams lie on the *xz*-plane (*φ_i_*_1_ = *φ_i_*_2_ = 0) so that Equation (4) becomes:

(6) $$\;\Phi =\frac{2\pi }{\lambda }\left[u\left(-\sin\theta_{i}\right)+w\left(1-\cos\theta_{i}\right)\right].$$

This relation can be used to calculate both the IP displacement u as well as the OP displacement **w** from the measured phase variation difference, assuming that only one type of displacements is occurring. In the cases where both types of displacements occur or when the displacement axis is unknown, an additional signal source for detection of IP displacement will be required, necessitating the combination of the aforementioned interferometer systems, as proposed in this paper.

In speckle interferometry, the non-mirror surface corresponds to the reflective grating of a reflective grating interferometer [9], with multiple beams incident on the rough surface instead of the grating, while the detector measures the intensity of the scattered light. However, unlike in grating interferometry, the light reflected by the speckle interferometer is scattered in all directions, therefore eliminating the need to align the detector to the reflected beam, although the signal strength will still vary depending on the location of the detector. Alignment of the direction of surface displacement is still necessary. As shown in Equation (3), the wavelength of the light source instead of the grating pitch is used to calculate the measured surface displacement.

### 2.2. Design of the Compound Speckle Interferometer System

This section covers the optical configuration of the proposed design. This compound speckle interferometer utilizes the relationship between the phase variations of the speckle interference patterns and the surface displacement as discussed above to obtain the displacement. This is achieved by acquiring interference signals from a combination of symmetrical and asymmetrical speckle interferometer systems, allowing for the measurement of both IP and OP displacements, as explained in detail below.

A heterodyne light source is obtained by passing a laser beam with a wavelength *λ* through a polarizer with a transmittance axis which is 45° to the horizontal plane. The electro-optical modulator (EOM) is modulated by a sawtooth signal at the preferred frequency. According to Su’s principle and Jones calculus [10], the electric field of the heterodyne light beam can be written as:

(7) $$E_{EOM}=\left[\begin{array}{c} e^{i\Delta \omega t/2}\\ e^{-i\Delta \omega t/2} \end{array}\right],$$

where Δ*ω* is the frequency of the modulation signal.

**Figure 3:**
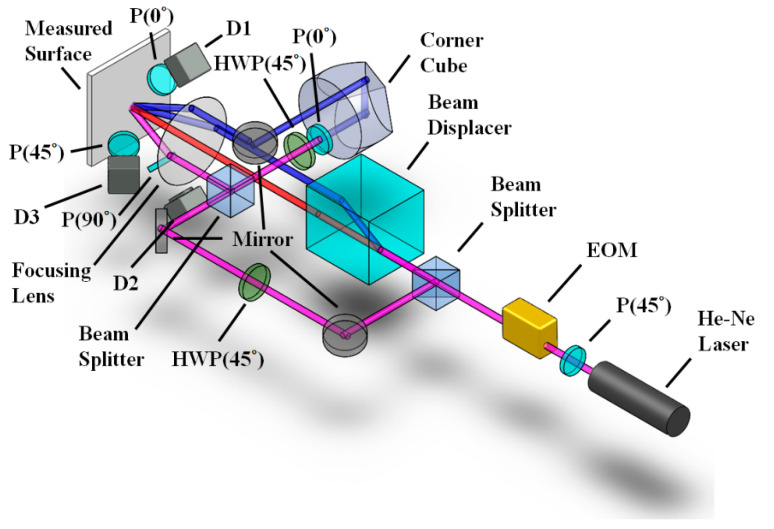
Diagram of the compound speckle interferometer configuration.

As shown in Figure 3, the heterodyne laser beam first enters a beam splitter and is split into two beams. The transmission beam then enters a beam displacer, which further splits the beam into two parallel beams in orthogonal polarization states, expressed here as *E_p_* and *E_s_*, with the respective electric field Jones vector representations shown here:

(8) $$E_{p}=BD_{p}\cdot E_{EOM}=\left[\begin{array}{c} e^{i\Delta \omega t/2}\\ 0 \end{array}\right],\;E_{s}=BD_{s}\cdot E_{EOM}=\left[\begin{array}{c} 0\\ e^{-i\Delta \omega t/2} \end{array}\right].$$

Meanwhile, the reflected beam, which is still polarized at 45° to the horizontal plane, is reflected again by a mirror and made to run parallel to the other two beams. It passes through a half-wave plate (HWP) placed at 45° to the azimuth, and the electric field becomes:

(9) $$E_{o}=HWP(45{}^{\circ})\cdot E_{EOM}=\left[\begin{array}{cc} 0 & 1\\ 1 & 0 \end{array}\right]\left[\begin{array}{c} e^{i\Delta \omega t/2}\\ e^{-i\Delta \omega t/2} \end{array}\right]=\left[\begin{array}{c} e^{-i\Delta \omega t/2}\\ e^{i\Delta \omega t/2} \end{array}\right].$$

This beam then passes through another beam splitter, forming two perpendicular beams. The beam traveling to the side passes through another 45° HWP and a 0° polarizer, reverting to *p*- and *s*-signals, before filtering the *s*-signals. After reaching the corner cube the beam is reflected back at an elevated height, before being reflected towards the surface by a mirror. The beam is parallel to the three beams below, and directly above the center beam. All four parallel beams are focused onto the measured surface by a focusing lens and scattered to form speckle interference. Each beam introduces a phase change into the resulting interference signal, which from Equation (3) we know is related to the incident angle of the beam. Assuming that the two side beams and the upper beam are equidistant from the center beam (assumed to be the *s*-beam here), the respective incident angles then are *θ_i_*_R_ = −*θ_iL_* = *θ_iU_* = *θ_i_*, *θ_iC_* = 0, and the resulting signals from each beam corresponding to Figure 3 are:

(10) $$\begin{array}{l} E_{R}=E_{p}\exp\left[i\left(\delta_{iR}+\delta_{oR}\right)\right]=\left[\begin{array}{c} e^{i\Delta \omega t/2}\\ 0 \end{array}\right]\exp\left\{i\frac{2\pi }{\lambda }\left[u\left(\sin\theta_{i}-\sin\theta_{oR}\right)+w\left(\cos\theta_{i}+\cos\theta_{oR}\right)\right]\right\}=\left[\begin{array}{c} e^{i\left(\delta_{R}+\Delta \omega t/2\right)}\\ 0 \end{array}\right],\\ E_{C}=E_{s}\exp\left[i\left(\delta_{iC}+\delta_{oC}\right)\right]=\left[\begin{array}{c} 0\\ e^{-i\Delta \omega t/2} \end{array}\right]\exp\left\{i\frac{2\pi }{\lambda }\left[u\left(-\sin\theta_{oC}\right)+w\left(1+\cos\theta_{oC}\right)\right]\right\}=\left[\begin{array}{c} 0\\ e^{i\left(\delta_{C}-\Delta \omega t/2\right)} \end{array}\right],\\ E_{L}=E_{o}\exp\left[i\left(\delta_{iL}+\delta_{oL}\right)\right]=\left[\begin{array}{c} e^{-i\Delta \omega t/2}\\ e^{i\Delta \omega t/2} \end{array}\right]\exp\left\{i\frac{2\pi }{\lambda }\left[u\left(-\sin\theta_{i}-\sin\theta_{oL}\right)+w\left(\cos\theta_{i}+\cos\theta_{oL}\right)\right]\right\}=\left[\begin{array}{c} e^{i\left(\delta_{L}-\Delta \omega t/2\right)}\\ e^{i\left(\delta_{L}+\Delta \omega t/2\right)} \end{array}\right],\\ E_{U}=P(0{}^{\circ})\cdot E_{p}\exp\left[i\left(\delta_{iU}+\delta_{oU}\right)\right]=\left[\begin{array}{c} e^{i\Delta \omega t/2}\\ 0 \end{array}\right]\exp\left\{i\frac{2\pi }{\lambda }\left[v\left(\sin\theta_{i}-\sin\theta_{oU}\right)+w\left(\cos\theta_{i}+\cos\theta_{oU}\right)\right]\right\}=\left[\begin{array}{c} e^{i\left(\delta_{U}+\Delta \omega t/2\right)}\\ 0 \end{array}\right], \end{array}$$

where *δ_R_*, *δ_C_*, *δ_L_* and *δ_U_* are the phase signals in relation to the incident and observation angles. 

For *x*-axis IP displacement, a detector (D1) with a 0° analyzer is used to receive the interference signal, as only *p*-polarized light from the right beam and left beam will be acquired, forming a symmetrical speckle optical configuration. For the *z*-axis OP displacement, a detector (D2) with a 90° analyzer is used to receive the interference signal and only *s*-polarized light from the center beam and left beam will be acquired, forming an asymmetric speckle optical configuration. For the *y*-axis IP displacement, a detector (D3) with a 45° analyzer is used to receive the interference signal, receiving all the signals. As such, the intensity signals received by the detectors *I*_1_, *I*_2_ and *I*_3_ will be:

(11) $$\begin{array}{l} I_{1}={\left|AN(0{}^{\circ})\cdot (E_{R}+E_{L})\right|}^{2}\propto \cos\left(\Delta \omega t+\frac{4\pi u}{\lambda }\sin\theta_{i}\right),\\ I_{2}={\left|AN(90{}^{\circ})\cdot (E_{R}+E_{C})\right|}^{2}\propto \cos\left(\Delta \omega t+\frac{2\pi }{\lambda }\left[-u\sin\theta_{i}+w\left(\cos\theta_{i}-1\right)\right]\right),\\ I_{3}={\left|AN(45{}^{\circ})\cdot (E_{R}+E_{L}+E_{C}+E_{U})\right|}^{2}\propto \cos\left(\Delta \omega t+\frac{2\pi u}{\lambda }\sin\theta_{i}+\frac{2\pi v}{\lambda }\sin\theta_{i}+\frac{4\pi w}{\lambda }\left(\cos\theta_{i}-1\right)\right). \end{array}$$

The phase variation of each signal Φ*_x_*, Φ*_z_* and Φ*_y_* can be extracted via a lock-in amplifier program:

(12) $$\begin{array}{l} \Phi_{x}=\frac{4\pi }{\lambda }u\sin\theta_{i},\\ \Phi_{z}=\frac{2\pi }{\lambda }\left[-u\sin\theta_{i}+w\left(\cos\theta_{i}-1\right)\right],\\ \Phi_{y}=\frac{2\pi }{\lambda }u\sin\theta_{i}+\frac{2\pi }{\lambda }v\sin\theta_{i}+\frac{4\pi }{\lambda }w\left(\cos\theta_{i}-1\right). \end{array}$$

From Φ*_x_* the *x*-axis IP displacement can be obtained, the *z*-axis OP displacement can be obtained from Φ*_z_* once the *x*-axis displacement is factored into the equation, and the *y*-axis IP displacement can be obtained from Φ*_y_* once the *x*-axis and *z*-axis displacements are factored into the equation.

## 3. Performance Tests and Discussion

The measurement capabilities and effectiveness of the proposed system were estimated with performance tests, including displacement measurement of different modes and ranges, repeatability and resolution tests on all axes, as well as measurement range tests.

**Figure 4:**
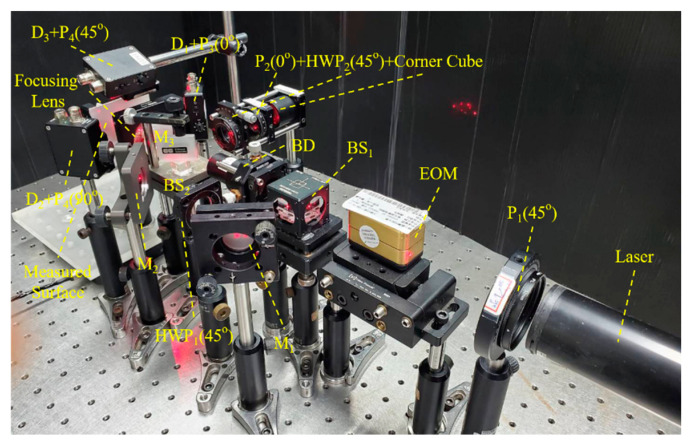
Compound speckle interferometer performance test setup.

The experimental setup for testing the measurement capabilities of the speckle interferometer is shown in Figure 4. For these tests, the light source consisted of a He-Ne laser (*λ* = 632.8 nm), a 45° polarizer, and an electro-optical modulator (EOM, Newport co., model: 4002) with the sawtooth heterodyne signal frequency set at 16 kHz. A piece of white cardboard was used as the measured sample because it presents a rough surface for speckle interferometry while reflecting a sufficient amount of light for detection and analysis. The white cardboard can also be replaced by other materials with a rough surface. To solve the problem of justification while the object was white cardboard, a CCD camera or quadrant detector can be utilized to sequentially ensure that the four laser beams were projected at the same point. Then, the white cardboard would be set on the position of the CCD camera. After completing the alignment of the white cardboard’s justification procedure, the process for adjusting the interference signals on the *x*-, *y*- and *z*-axis can then be performed. For long range displacement measurement, the cardboard sample was mounted on a long-range displacement stage (FS-1050XY, Sigma Koki, Tokyo, Japan), while for micrometer and nanometer range displacements a three-degree-of-freedom (3-DOF) precision positioning stage (P-562.3CD with a E-727.3CDA controller, both from Physik Instrumente, Karlsruhe, Germany) was used instead. For measurement signal acquisition, lock-in analysis and recording, photodetectors (PDA36A, Thorlabs, Newton, New Jersey, United States) were used to receive the interference signal. The data were sent for analysis (LabView, National Instruments, Austin, TX, USA) via a data acquisition (DAQ) card (National Instruments, PCI-6133 & BNC-2110). As for the reference signals, the long-range displacement platform has a built-in linear encoder, while the 3-DOF positioning stage has an internal capacitive sensor.

### 3.1. Displacement Tests for Various Ranges and Types of Movement

**Figure 5:**
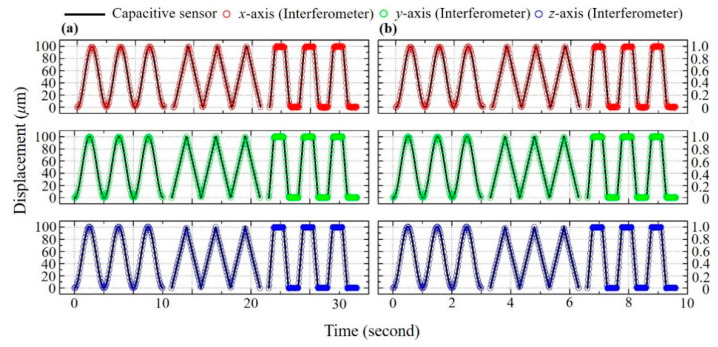
(**a**) Measurement results for 100 μm periodic displacements and (**b**) Measurement results for 1 μm periodic displacements.

In order to estimate the measurement capabilities of the proposed system for various modes of displacement, measurement tests were performed using different waveforms and different ranges. The medium and short-range displacement measurement tests were conducted with a 3-DOF positioning stage. Medium (100 μm) and short (1 μm) range sinusoidal, triangular, trapezoidal and random waveform displacements were performed on both the IP (*x*, *y*) and OP (*z*) axes. The experimental results were compared with the data acquired by the built-in capacitive sensor on the stage. The results are displayed below in Figure 5a,b. An examination of the figures shows that measurement data acquired by the compound speckle interferometer is similar to the data obtained from commercial capacitive sensors for the different modes of periodic movement. Besides, in order to verify the proposed system has high degree of linearity, we calculated the R-squared values of medium- and short-range triangular displacement signals on the *x*-, *y*- and *z*-axis are calculated and listed in the Table 1. It can be seen that the R-squared values are approximately 0.9998 to 0.9984, proving that the proposed system has high degree of linearity.

**Figure 6:**
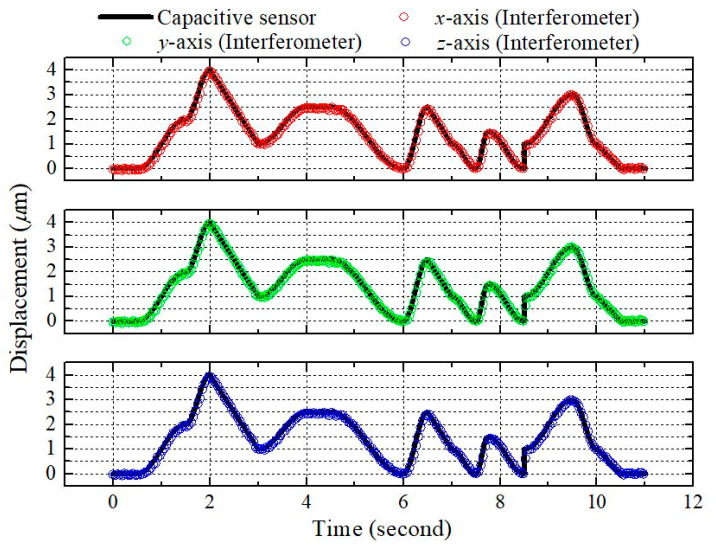
Measurement results for random displacement.

While the experiments discussed above verify the capability of our device for periodic displacement measurement, in practical applications, precision measurement of non-periodic displacement is also required. Therefore, to further test the capability of the proposed interferometer, a random displacement test was conducted with the stage and proposed system. Again, the results and the reference data were compared, as shown in Figure 6. The results show that the system is fully capable of random displacement measurement. The experiments demonstrate that the measurement capabilities of the proposed system are comparable those of commercially available precision displacement sensors.

### 3.2. Measurement Resolution and Repeatability Tests

According to the system specifications of the instruments used in the experiments, if one only considers the minimum interference signal which can be measured and analyzed using the signal analysis module in our system, the minimum phase variation which can be demodulated by our test equipment (National Instruments, PCI-6133, 14-Bit) and software lock-in program is approximately 0.022°, thus the “theoretical resolution” can then be calculated as approximately 0.111 nm accordingly, which would be the minimum displacement without considering the effects of disturbance. As such, it is necessary to determine the actual resolution of a measurement technique either by the minimum discernible displacement [6,11,12,13] or the discernible noise level that can be achieved or observed [14,15,16,17,18,19,20,21]. However, as indicated in the studies of Williamson et al. [14], Fleming et al. [15] and Lu et al. [16], if the measurement noise roughly corresponds to the Gaussian distribution, the resolution can be quantified by the standard deviation (σ) or root-mean-square (RMS) values of the noise level. Bobroff [17] also stated that resolution is the displacement equivalent of one standard deviation of the system noise. Moreover, Lee et al. [18] specified the measurement resolution to be the high-frequency oscillations attributed to the noise of the measurement system, which can be regarded as the minimum detectable measurement displacement. Therefore, in this study, the standard deviation (σ) of the noise level is utilized to estimate the measurement resolution of our proposed system.

**Figure 7:**
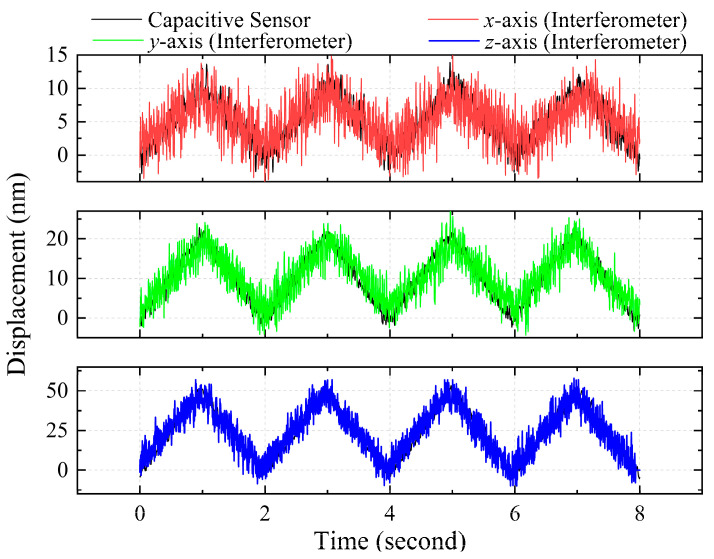
Measurement results of small triangular displacement tests.

To estimate the measurement resolution of our proposed system, small triangular displacement tests were performed. The positioning stage was driven to perform triangular wave motion displacements of 10 nm, 20 nm, and 50 nm on the *x*-, *y*-, and *z*-axis, respectively and the measurement results are plotted in Figure 7. Clearly, the measurement results of displacements along the *x*-, *y*-, and *z*-axis obtained using our method present a similarly high degree of linearity like those obtained using the capacitive sensor. As is apparent from the experiment results of the small triangular displacement tests, the displacement of approximately 10 nm on the *x*-axis can be clearly and consistently measured; simultaneously the discernible noise level of a few nanometers can also be clearly detected. In practice, the measured displacement is composed of the actual motion signal, as well as the low and high frequency noises. In general, low frequency noise results from background vibration and thermal drift while the high frequency noise comes from the laser light source, the photodetector, and the system’s electronics. Since the experiments in question involve a series of rapidly changing displacements within a short period of time, the effects of low frequency noise can be ignored. After calculating the experiment results of the small triangular displacement tests, the calculated standard deviation (σ) of the noise level on the *x*-axis is found to be approximately 2.03 nm, which can be regarded as the measurement resolution. Similarly, the calculated standard deviations (σ) of noise level on the *y*- and *z*-axis are found to be approximately 2.79 nm and 5.41 nm, which can be considered as the respective measurement resolutions. Additionally, it is worth noting that the calculated root-mean-square (RMS) values are also similar to the standard deviation (σ) values of the noise on each axis. As such, the standard deviation (σ) of the noise level is adopted as the measurement resolution of our proposed method, which can be found to be approximately 2.03 nm, 2.79 nm and 5.41 nm on the *x*-, *y*-, and *z*-axis after calculation.

Moving on, repeatability is another key performance index applicable to every measurement technique., its value can usually be evaluated or estimated through the calculation of the standard deviation (σ) for multiple rounds of experimentation [14,19,22,23,24,25]. For example, Chassagne et al. [19] performed a back and forth displacements of 5 mm for 20 times and the standard deviation (σ) of the positioning error (difference between starting and final positions) were calculated as 0.5 nm, which was regarded as the repeatability of their system. Therefore, in this study, the measurement repeatability is defined as the standard deviation (σ) of the difference between the average values of the starting position (origin) and the final position (return to origin) for 50 rounds. We find the mean (average) of the values of 400 sampling points at the starting and final positions, respectively and then subtract the two average values for each of the 50 rounds of experiments for calculating the standard deviation (σ). The smaller the standard deviation (σ) between the measured starting and final positions, the smaller the difference between each measurement, meaning better repeatability.

**Figure 8:**
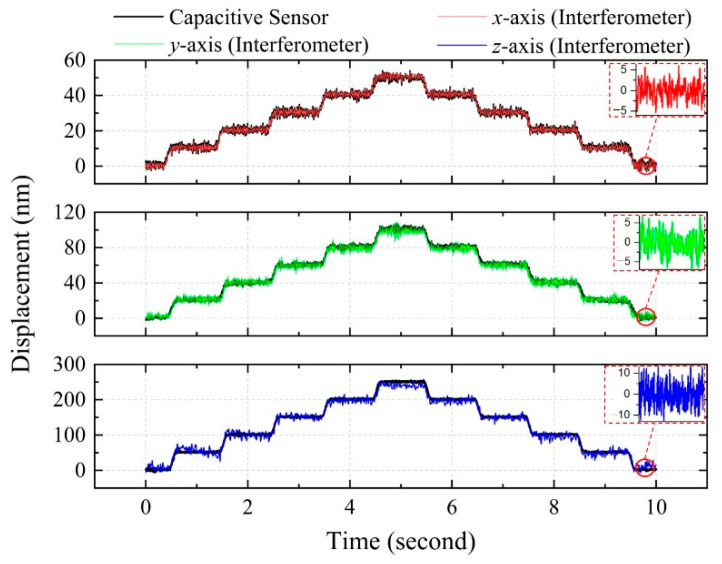
Measurement results of the step displacement tests.

In order to evaluate the repeatability of the proposed system, the positioning stage was set to move by 5 steps back and forth along each axis. As shown in Figure 8, the displacement on the *x* axis is 10 nm per step (red solid line); the displacement along the *y*-axis is 20 nm per step (green solid line) and the displacement along the *z*-axis is 50 nm per step (blue solid line). We applied the statistical method to evaluate the repeatability of measurement results, with the standard deviation (σ) of the measurement results for 50 step displacement tests, as demonstrated in the aforementioned studies, and the results on the *x*-, *y*-, and *z*-axis are shown in Table 2. It can be seen that the calculated standard deviation (σ) of the step displacement tests on the *x*-, *y*-, and *z*-axis are approximately 0.17 nm, 0.34 nm and 0.85 nm, respectively. 

Furthermore, according to our experiences, an increase in distance under the same travel speed means a longer period of time for the positioning stage to be in motion and for the system to conduct measurements may result in a change (and increase) in the repeatability value, since there is more time for environment disturbances to affect the measurement results. The research proposed by Li et al. [23] also indicates that the value of measurement repeatability is not a fixed constant, due to it being susceptible to the environmental disturbances. We also performed the step displacement experiments of 1 μm per step and 20 μm per step on each axis for 50 rounds to verify this phenomenon and the corresponding calculation value of the measurement results are also shown in Table 2. It can be seen from Table 2 that the calculated standard deviation (σ) for the two cases (1 μm/step and 20 μm/step) on each axis are less than 1.79 nm. This demonstrates the high measurement repeatability of the proposed compound speckle interferometer on each axis.

### 3.3. Evaluation of Measurement Repeatability through the Statistical Method of F-Test

In order to evaluate the significance of the measurement repeatability to the responses statistically through another method, the measurement results of step tests were examined with F-test for the studies of error distributions, as shown in the Table 2. Whereas the estimated means and confidence intervals were calculated at the 97.5% confident level (*α* = 2.5%). As a result, error sources from the system, data acquisition and parametric interaction contributed to the residuals and thereby the corresponding values of the repeatability and precision level of our system can be estimated. In this case, we repeatedly carried out the step experiments of 50 tests under the step back-and-forth motions on the *x*-axis with 10 nm per step for total 10 steps. In the statistical analysis [26], the absolute mean value of deviations between the starting and final positions is −0.02 nm for these 50 tests of the step experiments, with the calculated confidence intervals (CI) of ± 0.09. The equation can be expressed as:

(13) $$\mathrm{CI}=F_{\alpha ,\;r,\;n}\frac{s}{\sqrt{n}},$$

where *α* is the probability, *r* is the degrees of freedom for the numerator, *F* is the value of distribution, *s* is the sample standard deviation, *n* is the population of the test. Under the condition level of 97.5%, it can be known that *F*_0.025, 2, 50_ is 3.99 and *n* is 50. As a result, confidence interval (CI*_x_*) can be calculated as ±0.09 nm. Moreover, the other 50 tests of the step repeatability experiments with 20 nm and 50 nm per step on the *y*- and *z*-axis were also performed, respectively. After the same calculation procedures, the mean values of deviations between the starting and final positions for the *y*- and *z*-axis are 0.03 nm and 0.34 nm while the confidence interval CI*_y_* is ±0.19 nm and CI*_z_* is ±0.48 nm, respectively. Therefore, in this experiment, the measurement repeatability can also be regarded as the span of CI, which are 0.18 nm, 0.38 nm and 0.96 nm on the *x*-, *y*-, and *z*- axis, respectively. Moreover, the step displacement tests of 1 μm per step and 20 μm per step on each axis for 50 rounds were also been calculated and the corresponding calculation value of the span of CI are also shown in Table 2. Clearly, all the values of the span of CI and the calculated standard deviation (σ) are close, displaying a high degree of consistency between the two methods. Therefore, we can regard these values as the measurement repeatability on the *x*-, *y*-, and *z*-axis corresponding to the respective displacement ranges, regardless of whether the span of CI or the standard deviation is utilized to estimate the measurement repeatability.

### 3.4. Discussion of Estimated Confidence Intervals and Standard Deviations

In this work, the measurement uncertainty would involve errors from the system, data acquisition and parametric interactions, which can usually be resolved by the analysis of residuals, Daniel half-normal plots and two-way ANOVA. In our work, the error values collecting from the system, data acquisition and parametric interactions are quantitatively collected and presented by the estimated standard deviations. When the calculated standard deviations are within the estimated confidence intervals with appreciable values, the statistical analysis for resolving the errors from the system, data acquisition and parametric interaction would not be urged to carry out. 

Fundamentally, if the mean standard deviations were converged in comparisons with their corresponding confidence intervals under a F distribution, the precision level would be acceptable. Hence, a listed table to show the estimated CI and the standard deviation would be sufficient. If the calculated standard deviations were greater than that of confidence intervals in the repetition tests, then investigations of error sources in the aspects of the set-up system, data acquisition and parametric interactions will be revisited.

Consequently, in the case of the step tests with 20 μm per step, we firstly use standard deviation to estimate the measurement uncertainty of our proposed method. Secondly, we calculated the confident intervals from the experimental results of the step tests. Since the calculated confidence intervals of ±0.56 nm, ±0.57 nm, and ±1.01 nm on the *x*-, *y*-, and *z*-axis respectively were greater than the mean standard deviations, with 97.5% confidence level, meaning that the standard deviations of the measurement errors on the *x*-, *y*-, and *z*-axis of approximately 0.98 nm, 1.01 nm, and 1.79 nm are sufficient to demonstrate the precision level of the system, instead of the uncertainties from the error sources.

### 3.5. Measurement Results of Straightness

**Figure 9:**
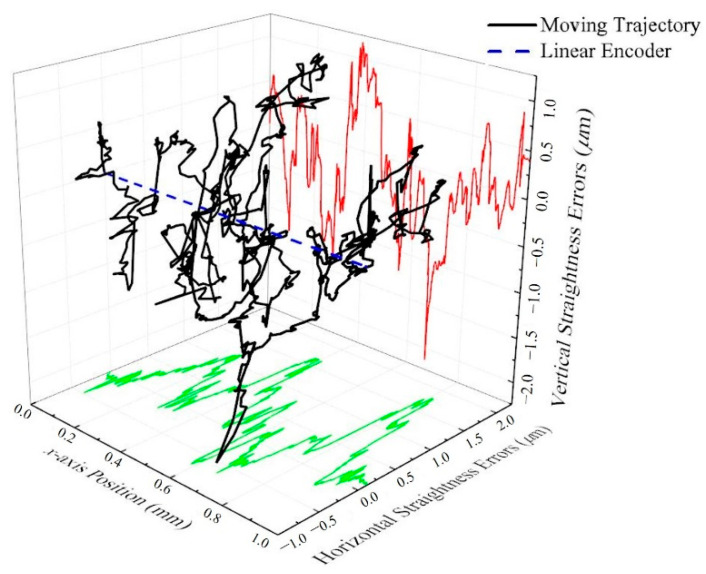
Straightness error measurement test results.

In order to verify the proposed system is capable of measuring 3-DOF displacement simultaneously, a straightness error measurement experiment for a travel range of 1 mm along the *x*-axis was conducted. In this experiment, we use a processed aluminum block (Surface roughness Ra: 2.06 μm) as the measured object to prevent the surface from being flat enough to affect the measurement results. Then, the stage was moved along the *x*-axis while the measurement results of our proposed method on the *x*-, *y*-, and *z*-axis were utilized to provide the information of positioning, vertical straightness, and horizontal straightness, respectively, which is shown in Figure 9. It can be seen that the blue dotted line represents the displacement conducted by the linear encoder; while the black solid line represents the trajectory of the straightness, which the value of vertical straightness is projected onto the right-hand surface with red solid line, and the value of horizontal straightness is projected onto the bottom surface with green solid line. It can be known from the results that the vertical straightness is within 2.14 μm and the horizontal straightness is within 1.81 μm, respectively, proving that the proposed system can provide not only the displacement but also the vertical (*y*-axis) and horizontal (*z*-axis) straightness simultaneously with the object surface of different materials.

The proposed system is capable of measuring most materials with rough surfaces. In the displacement, resolution, repeatability, and maximum measurement range tests, a white cardboard was used as the measured object because the speckle interference patterns (signals) can be detected in all directions when the laser is projected onto the surface of the white cardboard and then scattered. The white cardboard was fixed by a mounting plate and set on the positioning stage. If the stage moves along two or more directions simultaneously, the measurement results might be influenced by the surface flatness of the white cardboard. Hence, the white cardboard was replaced with a processed aluminum block to prevent the surface from being flat enough to affect the measurement results when the 3-DOF straightness error measurement test was performed.

### 3.6. Maximum Measurement Range and Dynamic Range

**Figure 10:**
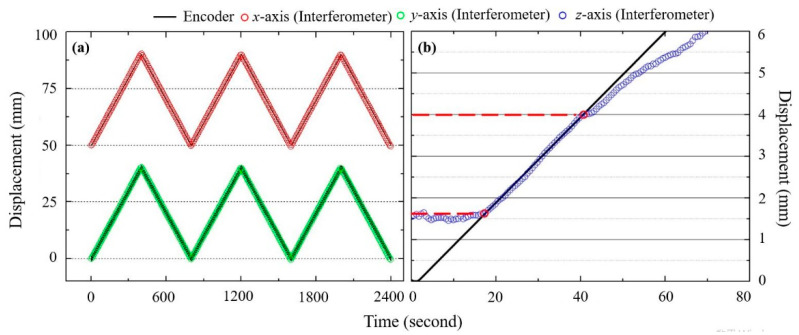
(**a**) IP displacement measurement range test results; (**b**) OP displacement measurement range test results.

Owing to their non-contact mode of measurement, speckle interferometers technically have an unlimited range when it comes to IP displacement measurement. In practice, however, the maximum measurement distance is still limited by the measurement surface and the positioning stage itself. In order to determine the consistency for IP displacement measurement over long distances, the long-range displacement platform is set to travel a distance of 40 mm, the longest stable displacement the platform is capable of, along the IP axes *x* and *y*. The proposed system was set to measure for an entire stroke. A comparison of the results with those obtained with the platform’s built-in linear encoder is shown in Figure 10a. The results demonstrate that the system is fully capable of accurately measuring long-range displacements along both IP axes.

In the case of OP displacement, there is a limit to the measurement range of the system. As the measured surface away from the focal point of the laser beams, defocusing errors gradually accumulate which affects the results. The theoretical measurement range of the interferometer therefore depends on the size of the overlap of the laser beams on the measured surface. To better understand the limits of the system, OP displacement measurement range testing was conducted, with the results compared with data from the stage’s built-in encoder for reference. As can be seen in Figure 10b, the maximum distance where the measured displacement is reasonably consistent with the reference data is around 3.4 mm, after which measurement errors become too pronounced. Theoretically, the OP measurement range can be improved in two ways, either by expanding the laser beams to increase the area of overlap but degrading the speckle interference signal, or by decreasing the incident angles, to ensure that the beams stay overlapped for longer ranges, at the cost of the measurement resolution, according to Equations (5) and (6).

Moreover, the dynamic range is defined as the total displacement range divided by the measurement resolution [27,28]. According to the experiment results of the maximum measurement range and resolution tests, the measurement ranges along the *x*-, *y*-, and *z*-axis are divided by the corresponding measurement resolutions, then it can be established that the dynamic ranges of the proposed system on the *x*-, *y*-, and *z*-axis are 4 × 10^6^, 2 × 10^6^ and 6.8 × 10^4^, respectively.

### 3.7. Limitations: Speed of Measurement

In our proposed system, the measurement speed was limited mainly by the lock-in amplifier (LIA) software because the optical modulation frequency was higher than the calculation rate (*f*_C_) of the LIA software. In theory, the relationships among the phase difference variation rate (*d*Φ*/dt*), measurement sensitivity, and measurement speed (*d(d_x_)/dt*) can be written as (*d*Φ*/dt = s × d(d_x_)/dt*). To prevent a loss of data, the phase difference variation rate should be lower than the calculation rate (*f*_C_) of the LIA software; i.e., *d*Φ*/dt = s* × *d(d_x_)/dt* < *π* × *f*_C_. Obviously, the calculation rate (*f*_C_) of the LIA software limits the speed of measurement. In our current set-up, the *f*_C_ in the measurement speed test was approximately 1000 Hz (1 ms). In this study, the sensitivity (*S*) of the *x*-, *y*- and *z*-axis is 1.0972 *π*/μm, 0.5487 *π*/μm and 0.0481 *π*/μm, while the speed limit is 1822.8 μm/s, 3645.0 μm/s and 41,580.0 μm/s, respectively under the same calculation rate (*f*_C_). We then conducted experiments that verify that the speed limits along the *x*- and *y*-axis are consistent with the theoretical values. However, due to the limited measurement range for the *z*-axis (3.4 mm) and the inherent maximum speed of our positioning stage (41,580.0 μm/s), so the measurement speed limit along the *z*-axis cannot be verified. Nevertheless, according to our previous experience, the theoretical value of the speed limits along the *x*- and *y*-axis are consistent with the actual value, so we can deduce that the actual speed limit along the *z*-axis should also reach its theoretical value.

### 3.8. Analysis of the Periodic Nonlinear Error

The interferometer measurement results often include non-linear periodic errors which occur as a consequence of imperfections in the polarization of the optical components. The primary sources of errors within the proposed system are component misalignments (frequency mixing), and the extinction ratio inherent to optical components (polarization mixing). In this section, the sources and effects of non-linear periodic errors for each measured dimension are estimated and analyzed.

The main contributors of non-linear errors to this system are the beam displacer, polarizers (including the analyzers for separating signals) and HWPs. To estimate the non-linear errors, the previous calculations are run again, but this time accounting for the aforementioned error sources, substituting their altered Jones matrices into the equations:

(14) $$\begin{array}{l} E_{p}{}^{*}=BD_{p}\left(\varepsilon_{BD},\theta_{BD}\right)\cdot E_{EOM},\;E_{s}{}^{*}=BD_{p}\left(\varepsilon_{BD},\theta_{BD}\right)\cdot E_{EOM},\\ E_{o}{}^{*}=HWP_{1}\left(45{}^{\circ}+\theta_{h1}\right)\cdot E_{EOM},\;E_{u}{}^{*}=P\left(\varepsilon_{P},\theta_{P}\right)\cdot HWP_{2}\left(45{}^{\circ}+\theta_{h2}\right)\cdot E_{o}{}^{*}, \end{array}$$

where *ε* is the extinction ratio and *θ* is the azimuth angle misalignments corresponding to the components. Now, the resulting signals can be calculated. The resulting signal each photodetector receives is:

(15) $$\begin{array}{l} E_{m1}=AN\left(\varepsilon_{AN1},\theta_{AN1}\right)\left[E_{p}{}^{*}e^{i\delta_{R}}+E_{o}{}^{*}e^{i\delta_{L}}\right],\\ E_{m2}=AN\left(\varepsilon_{AN2},90{}^{\circ}+\theta_{AN2}\right)\left[E_{s}{}^{*}e^{i\delta_{C}}+E_{o}{}^{*}e^{i\delta_{L}}\right],\\ E_{m3}=AN\left(\varepsilon_{AN3},45{}^{\circ}+\theta_{AN3}\right)\left[E_{s}{}^{*}e^{i\delta_{C}}+E_{u}{}^{*}e^{i\delta_{U}}\right]. \end{array}$$

The corresponding intensity signals received by the photodetectors can be represented by the following general equation:

(16) $$\;I={\left|E_{m}\right|}^{2}\propto AC\cos\left(\omega t+\Phi {}^{\prime }\right),$$

where Φ′ stands for the phase affected by non-linear errors. The corresponding phase errors Φ_error_ can be obtained from the following general equation:

(17) $$\Phi_{error}=\Phi {}^{\prime }\left(\varepsilon_{BD},\theta_{BD},\theta_{h1},\theta_{h2},\varepsilon_{P},\theta_{P},\varepsilon_{AN1},\theta_{AN1},\varepsilon_{AN2},\theta_{AN2},\varepsilon_{AN3},\theta_{AN3}\right)-\Phi .$$

The displacement errors can be calculated from the preceding phase errors. However, as the derived *z*-axis displacement value is dependent on the measured *x*-axis displacement and the derived *y*-axis displacement value is in turn dependent on the *x*- and *z*-axis displacements, the measured displacements will also be affected by the preceding phase errors. Thus, the displacement errors can be expressed by:

(18) $$u_{error}=\frac{\Phi_{x_{error}}\lambda }{4\pi \sin\theta_{i}},\;w_{error}=\frac{\left(\Phi_{x_{error}}+2\Phi_{z}{}_{{}_{error}}\right)\lambda }{4\pi \left(\cos\theta_{i}-1\right)},\;v_{error}=\frac{\left(2\Phi_{y_{error}}-3\Phi_{x_{error}}-4\Phi_{z}{}_{{}_{error}}\right)\lambda }{4\pi \sin\theta_{i}}.$$

In the case of our setup, the minimum adjustment angles for the polarizers and HWPs could be controlled to within 5 by the use of precision rotation mounts, while the adjustable platform carrying the beam displacer is estimated to have an error range of approximately 1°. The extinction ratios are 1:20,000 for the beam displacer and 1:4000 for the polarizers, respectively. By substituting the above parameters into the general equation, Equation (16), the non-linear periodic errors for the three types of displacement are estimated to be about 0.85°, 1.57° and 7.88° for axes *x*, *y* and *z*, respectively. Assuming the incident angles *θ_i_* of all the beams to be 45°, the displacement errors of the three axes can be further obtained from Equation (17), turning out to be 1 nm, 38.4 nm and 49.8 nm for *x*, *y* and *z*, respectively. However, if we only factor in the extinction ratios of the components, their effects on non-linear errors are disproportionately small. Therefore, we can conclude that for the proposed speckle interferometer, the dominating factor for non-linear errors is the azimuth angle misalignment of the polarizing components. The obvious way to eliminate non-linear periodic errors in the system would be to utilize more precise component holders to limit azimuth angle misalignment errors. Another way would be to increase the incident angle of the beams, as can be inferred from Equation (17), which would have the additional benefit of increasing the resolution, as demonstrated in Equations (5) and (6), but the drawback of decreasing the OP displacement measurement range, as we discovered above.

### 3.9. Comparison with State-of-Art Interferometers

First, regarding comparisons with existing studies, it is known there are many kinds of interferometers developed for the purposes of displacement measurement, rotation angles measurement, or system calibration (geometric error measurement). Since the proposed method is developed for displacement measurement and system calibration, several state-of-art methods of displacement measurement or system calibration are listed in Table 3 for comparison. As can be seen from the table, the method proposed by Lee et al. [12] is capable of measuring 1-DOF displacement with the resolution and repeatability of 2 nm and 2 nm, respectively. The measurement system is compact consisting of only a few optical components and one photodetector. However, the measuring range is limited to a few microns. By utilizing a beam-splitting, the method proposed by Wu et al. [29] is able to achieve 1-DOF displacement and 2-DOF rotation measurements with the respective resolutions of 0.8 nm and 0.4”. Nevertheless, since the working principles of Lee’s and Wu’s systems are designed based on the wavelength of the light source, measurement results might be affected by unstable wavelengths caused by thermal drift.

To overcome problems associated with unstable light wavelength, grating-type interferometers were developed. Hsieh et al. [13] developed a heterodyne Wollaston grating interferometer with a common-optical-path configuration for the measurement of in-plane displacements. Experiment results reveal that Hsieh’s method is capable of sensing 1-DOF displacement to a resolution of 2 nm while maintaining the stability of the system against environmental disturbances. Lv et al. [30] proposed a grating interferometer which based its design concept on the Littrow optical configuration, 2-DOF in-plane and out-of-plane displacements can be achieved with the resolution and repeatability of 0.27 nm and 2 nm, respectively. By adopting a similar design concept with two position sensitive detectors, the method proposed by Qiang et al. [31] is able to provide the information of 2-DOF in-plane and out-of-plane displacements and 3-DOF rotations with the resolutions of 4 nm and 0.2”, respectively. However, none of the grating interferometers mentioned above are able to provide simultaneous 2-DOF in-plane displacement information unless a 2D grating or a pair of orthogonal 1D gratings are used in the system. This presents several complications in terms of system design and application, as it is essential for the grating pair to be arranged completely orthogonal to one another to ensure measurement accuracy, while a 2D grating with a large detection area is difficult to acquire through conventional means. 

As for the development of commercial interferometers, Renishaw Company [32,33] and Keysight Company [34] have proposed different interferometers for displacement measurement or system calibration. As can be seen from Table 3, Renishaw’s interferometer can achieve respective measurement resolutions of 1 nm and 0.03 μrad for displacement and rotation while the resolution of Keysight’s interferometer is 0.25 nm for displacement.

In contrast, our system combines the techniques of speckle interferometry and heterodyne interferometry to effectively alleviate the problem of low interference contrast of speckle patterns, and thus the 3-DOF displacements of a measured surface can be obtained through direct analysis of the corresponding heterodyne interference signals, with the resolutions of 10 nm, 20 nm, and 50 nm on the *x*-, *y*-, and *z*-axis, respectively. Compared with the measurement techniques and systems mentioned above, the proposed system only requires a surface with a flat, slightly rough surface for accurate measurements in multiple DOFs, and is not limited by factors such as an optical ruler, which is conductive for the measurement large displacements. Also, the system configuration does not require precise mirror positioning or grating components, greatly reducing system complexity and cost. Additionally, since the speckle interference patterns (signals) from the laser beams scattered by the measured surface can theoretically be detected from any direction, the detector positions are not fixed relative to the system itself, only required to be stationary during measurement for consistent results, the system setup is very flexible and can be adapted for a variety of situations. All these are features that we believe are the advantages of our proposed system.

The motivation of our proposed method is to develop a potential interferometer which can be used to the application fields of precision machinery industry or machine tools industry for displacement measurement or system calibration. For example, the XL80 interferometer provided by Renishaw Company was developed to achieve displacement measurement for positioning stage while the XM60 interferometer was developed to perform system calibration for CNC machine tools. As can be seen from the successful experiments in this study, the proposed method is capable of providing the information of 3-DOF displacements or geometric errors with nano-meters level resolution for positioning stages simultaneously without changing its optical arrangement. Additionally, the proposed system can further simplify the 3-DOF configuration into 2-DOF or 1-DOF according to the requirements for different measurement purpose. Hence, in our opinion, these are the potential application fields of our proposed method.

## 4. Conclusions

In this study, a compound speckle interferometer for measuring 3-DOF displacement is developed. The system which incorporates design concepts from heterodyne interferometry, speckle interferometry and beam splitting techniques can accurately measure the IP and OP displacement of a non-mirror surface. Speckle interference is formed by having multiple beams converge on the same point of the measured surface. The phase of the interference pattern will shift according to the surface displacement, allowing for precision displacement measurement. Through the use of polarizers and an HWP to separate and filter the displacement signals on three axes, the system is capable of simultaneous 3-DOF displacement measurement. Several tests were conducted to verify the measurement capabilities of the system, the calculated standard deviations (σ) of noise levels are 2.03 nm, 2.79 nm and 5.41 nm on the *x*-, *y*-, and *z*-axis, respectively, which can be regarded as the measurement resolution on the corresponding axis of our system. Moreover, the measurement repeatability was estimated through the calculated standard deviation (σ) or the span of confidence interval (CI) from the step tests. In the case of the 20 μm per step, the calculated standard deviation (σ) can be found to be approximately 0.98 nm, 1.01 nm, and 1.79 nm on the *x*-, *y*-, and *z*-axis, respectively, which are regarded as the measurement repeatability. Meanwhile, the calculated values of the standard deviation (σ) and the span of CI for on each axis are also close, displaying a high degree of consistency between the two analysis methods. The experimental results are compared with measurements from the internal displacement sensors within the stages. It is shown that the measurement capability of the proposed system is comparable to that of commercial precision displacement sensors. Compared with contemporary precision displacement measurement techniques, this system has the advantages of being able to conduct simultaneous 3-DOF displacement measurement, being a non-contact-based method as well as possessing high resolution, excellent versatility and long measurement range. 

## Figures and Tables

**Table 1:** R-squared values of medium- and short-range triangular displacement signals on the *x*-, *y*-, and *z*-axis.

Direction	100 μm	1 μm
*x* axis	0.9995	0.9989
*y* axis	0.9998	0.9991
*z* axis	0.9984	0.9984

**Table 2:** Calculation results of the step tests.

Direction	Range/Step	Mean Value	Standard Deviation (σ)	CI	Span of CI
*x* axis	10 nm	−0.02 nm	0.17 nm	±0.09 nm	0.18 nm
*y* axis	20 nm	−0.03 nm	0.34 nm	±0.19 nm	0.38 nm
*z* axis	50 nm	0.34 nm	0.85 nm	±0.48 nm	0.96 nm
*x* axis	1 μm	0.29 nm	0.53 nm	±0.30 nm	0.60 nm
*y* axis	1 μm	0.24 nm	0.60 nm	±0.34 nm	0.68 nm
*z* axis	1 μm	0.75 nm	0.95 nm	±0.53 nm	1.06 nm
*x* axis	20 μm	−0.71 nm	0.98 nm	±0.56 nm	1.12 nm
*y* axis	20 μm	−1.38 nm	1.01 nm	±0.57 nm	1.14 nm
*z* axis	20 μm	2.35 nm	1.79 nm	±1.01 nm	2.02 nm

**Table 3:** Comparison with state-of-art interferometers.

Year/Author/ Reference	Measurement Degree	Measurement Range (max)	Resolution	Repeatability	Measurement Speed
2020 Lee et al. [12]	1-DOF	10 μm	2.5 nm	Not provide	Not provide
2017 Wu et al. [29]	3-DOF	50 mm	*z*: 0.8 nm *θ_pitch_*: 0.4” *θ_yaw_*: 0.5”	Not provide	Not provide
2018 Hsieh et al. [13]	1-DOF	20 mm	2 nm	1 nm	1100 μm/s
2018 Lv et al. [30]	2-DOF	10 mm	*x*: 0.27 nm *z*: 0.18 nm	*x*: 2 nm *z*: 2 nm	Not provide
2020 Qiang et al. [31]	5-DOF	Not provide	*x*: 4 nm *z*: 4 nm *θ_pitch_*: 1′’ *θ_yaw_*: 1′’ *θ_roll_*: 1′’	Not provide	Not provide
2016 Renishaw XL-80 [32]	1-DOF	80 m	1 nm	Not provide	4 m/s
2017 Renishaw XM-60 [33]	6-DOF	4 m	*x*: 1 nm *y*: 1 nm *z*: 1 nm *θ_yaw_*: 0.03 μrad *θ_pitch_*: 0.03 μrad *θ_roll_*: 0.12 μrad	Not provide	1 m/s
2017 Keysight 5530 [34]	3-DOF	30 m	0.25 nm	Not provide	1 m/s
Proposed Interferometer	3-DOF	40 mm	*x*: 2.03 nm *y*: 2.79 nm *z*: 5.41 nm	*x*: 0.98 nm *y*: 1.01 nm *z*: 1.79 nm	*x*: 1.82 mm/s *y*: 3.65 mm/s *z*: 41.58 mm/s

## Data Availability

All data of this study can be provided through e-mail request.
